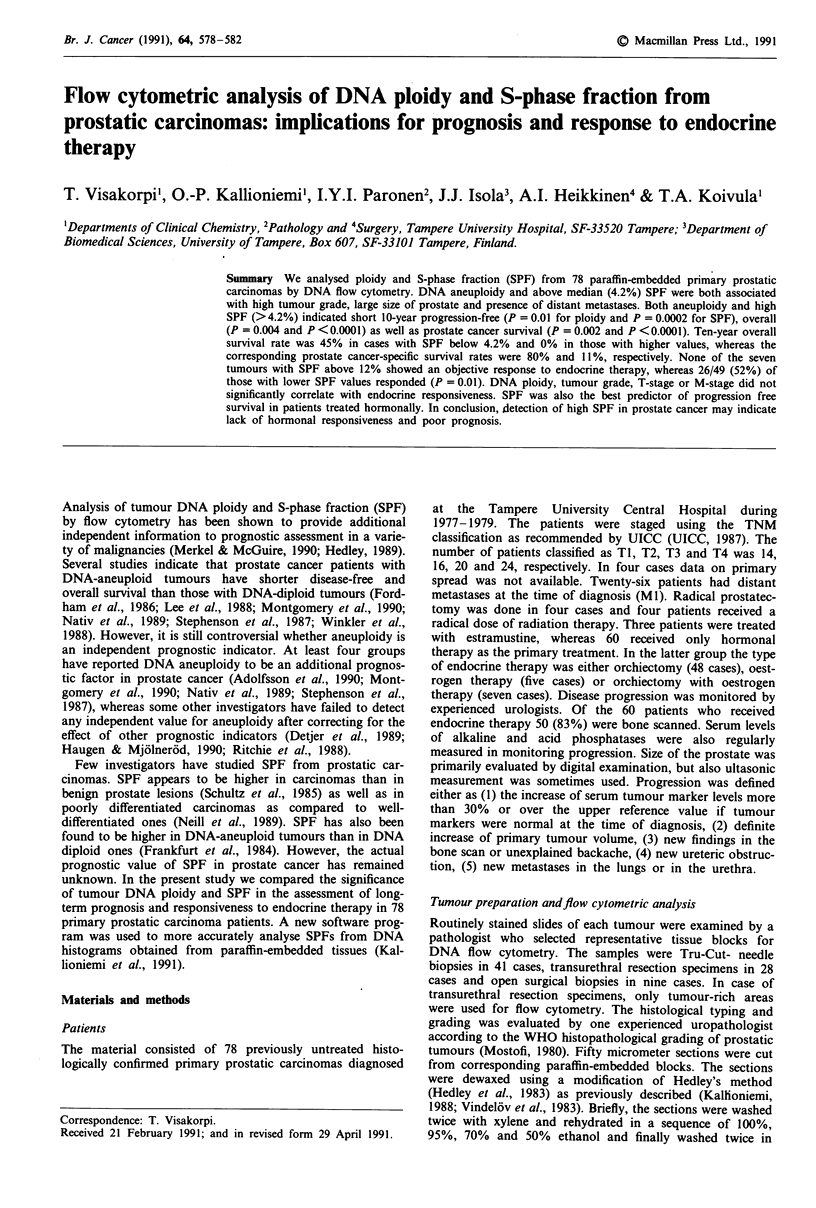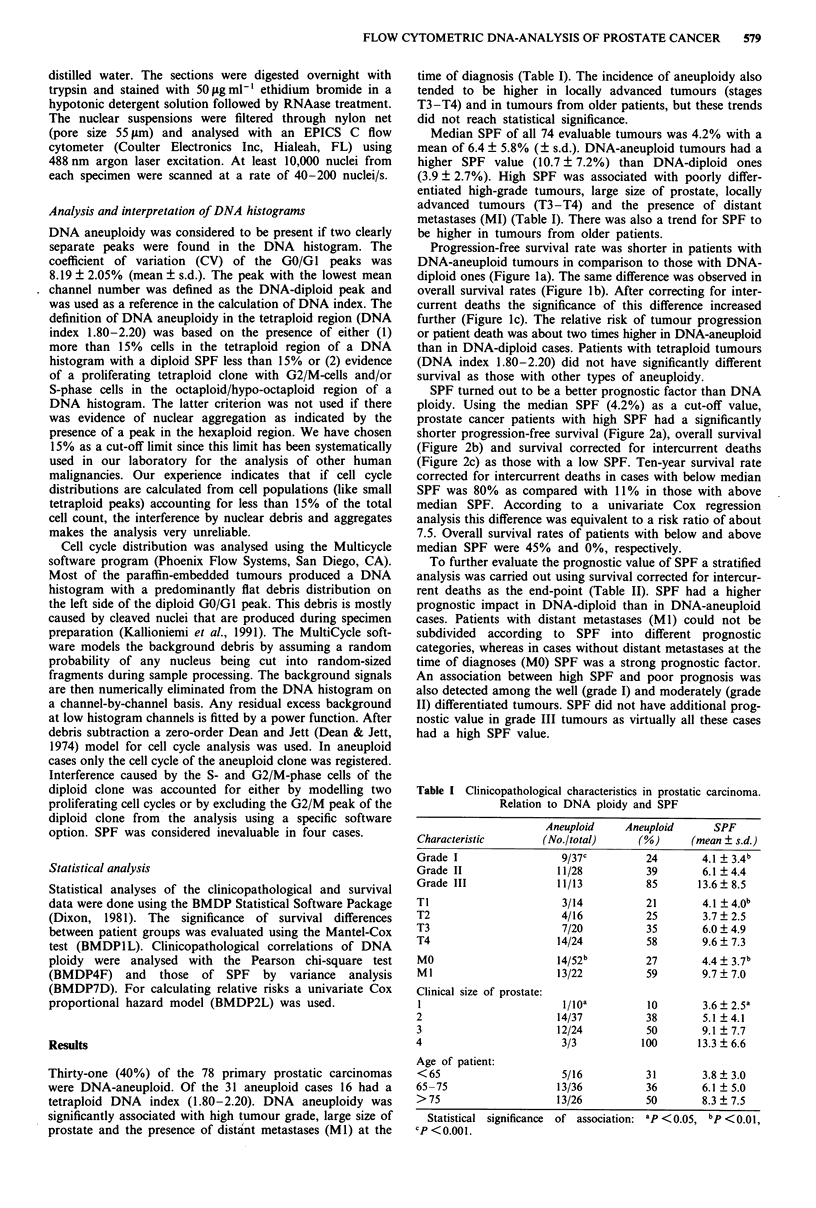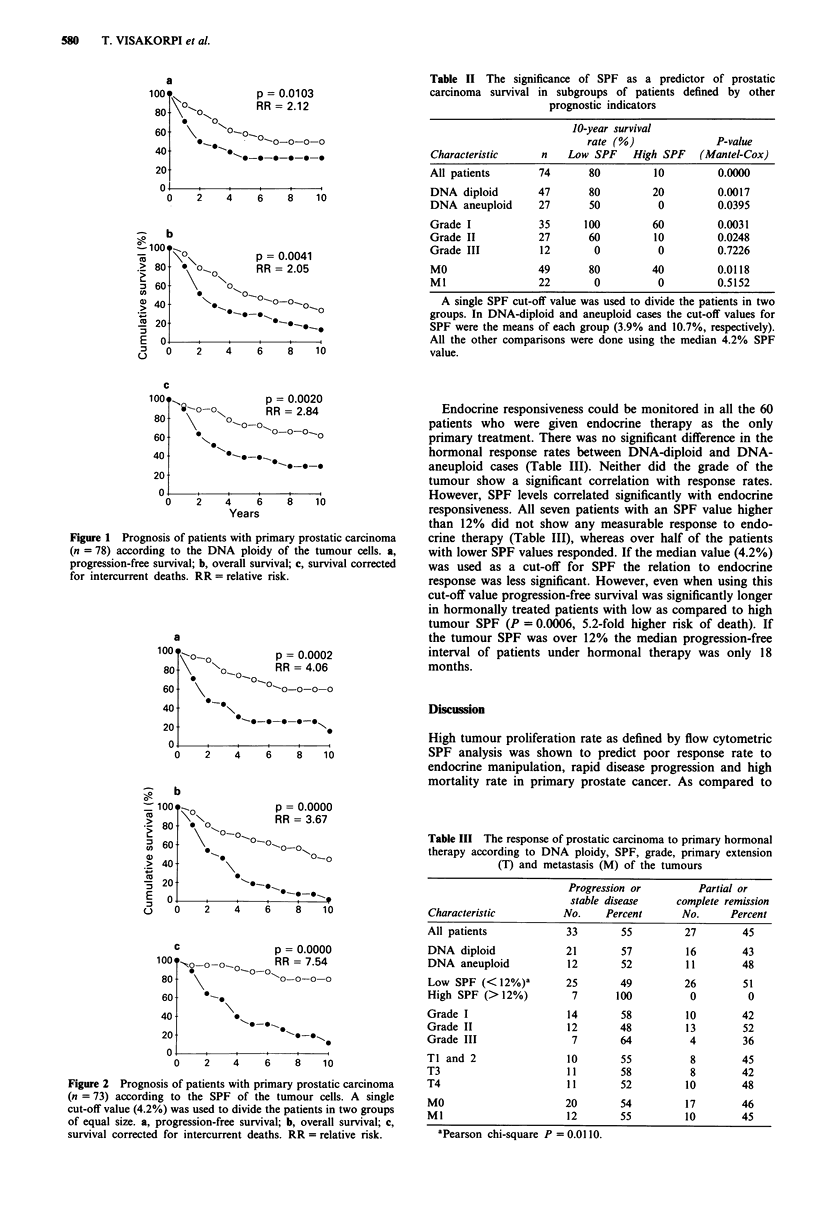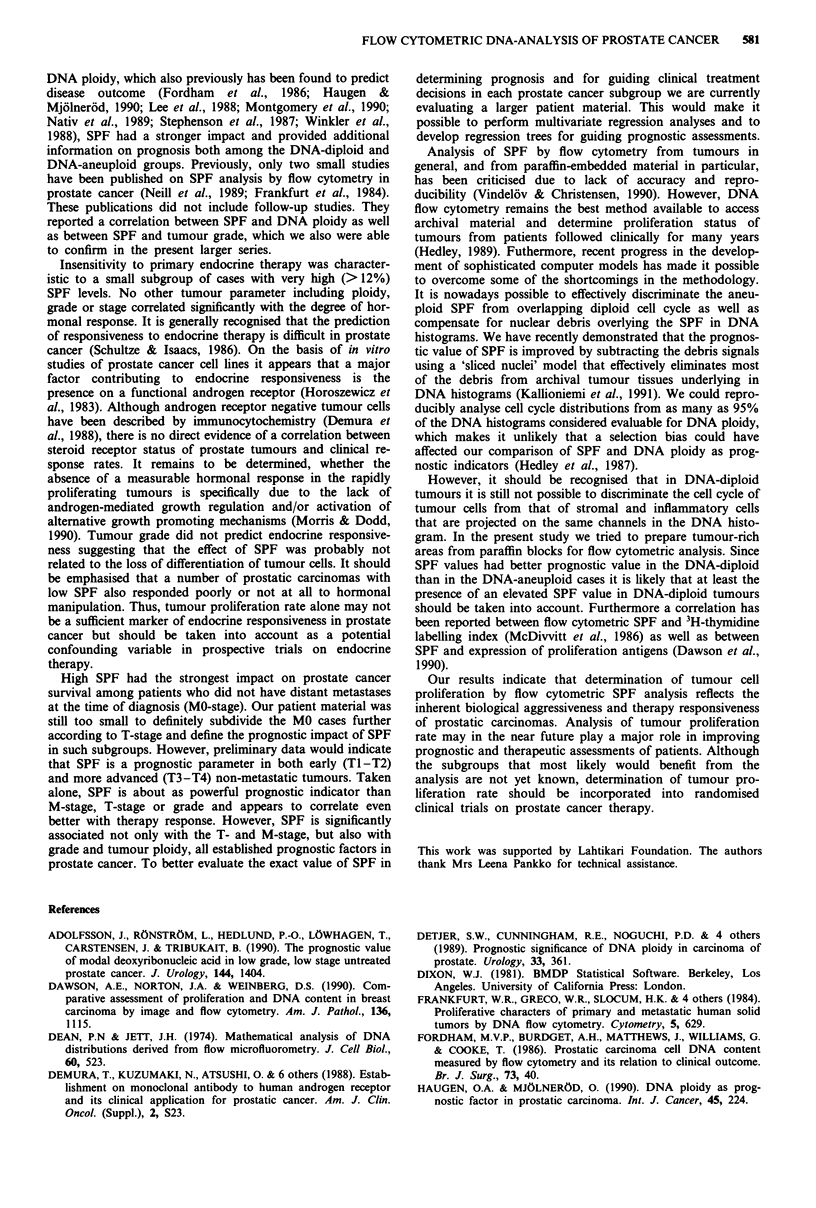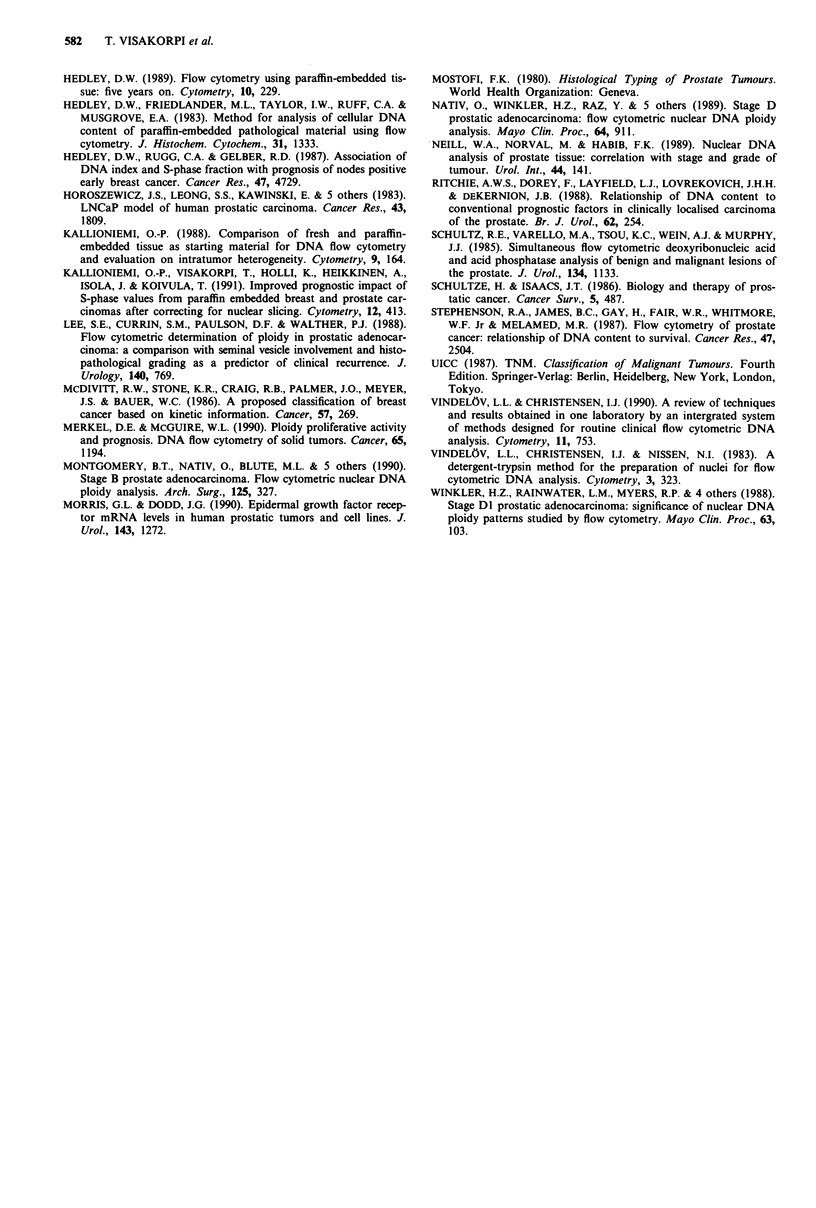# Flow cytometric analysis of DNA ploidy and S-phase fraction from prostatic carcinomas: implications for prognosis and response to endocrine therapy.

**DOI:** 10.1038/bjc.1991.353

**Published:** 1991-09

**Authors:** T. Visakorpi, O. P. Kallioniemi, I. Y. Paronen, J. J. Isola, A. I. Heikkinen, T. A. Koivula

**Affiliations:** Department of Clinical Chemistry, Tampere University Hospital, Finland.

## Abstract

We analysed ploidy and S-phase fraction (SPF) from 78 paraffin-embedded primary prostatic carcinomas by DNA flow cytometry. DNA aneuploidy and above median (4.2%) SPF were both associated with high tumour grade, large size of prostate and presence of distant metastases. Both aneuploidy and high SPF (greater than 4.2%) indicated short 10-year progression-free (P = 0.01 for ploidy and P = 0.0002 for SPF), overall (P = 0.004 and P less than 0.0001) as well as prostate cancer survival (P = 0.002 and P less than 0.0001). Ten-year overall survival rate was 45% in cases with SPF below 4.2% and 0% in those with higher values, whereas the corresponding prostate cancer-specific survival rates were 80% and 11%, respectively. None of the seven tumours with SPF above 12% showed an objective response to endocrine therapy, whereas 26/49 (52%) of those with lower SPF values responded (P = 0.01). DNA ploidy, tumour grade, T-stage or M-stage did not significantly correlate with endocrine responsiveness. SPF was also the best predictor of progression free survival in patients treated hormonally. In conclusion, detection of high SPF in prostate cancer may indicate lack of hormonal responsiveness and poor prognosis.


Br. J. Cancer (1991), 64, 578–582 © Macmillan Press Ltd., 1991

### Flow cytometric analysis of DNA ploidy and S-phase fraction from prostatic carcinomas: implications for prognosis and response to endocrine therapy

T. Visakorpi[1], O.-P. Kallioniemi[1], I.Y.I. Paronen[2], J.J. Isola[3], A.I. Heikkinen[4] & T.A. Koivula[1]

[1]*Departments of Clinical Chemistry,* [2]*Pathology and* [4]*Surgery, Tampere University Hospital, SF-33520 Tampere;* [3]*Department of Biomedical Sciences, University of Tampere, Box 607, SF-33101 Tampere, Finland.*

**Summary** We analysed ploidy and S-phase fraction (SPF) from 78 paraffin-embedded primary prostatic carcinomas by DNA flow cytometry. DNA aneuploidy and above median (4.2%) SPF were both associated with high tumour grade, large size of prostate and presence of distant metastases. Both aneuploidy and high SPF (>4.2%) indicated short 10-year progression-free ($P$ = 0.01 for ploidy and $P$ = 0.0002 for SPF), overall ($P$ = 0.004 and $P$ < 0.0001) as well as prostate cancer survival ($P$ = 0.002 and $P$ < 0.0001). Ten-year overall survival rate was 45% in cases with SPF below 4.2% and 0% in those with higher values, whereas the corresponding prostate cancer-specific survival rates were 80% and 11%, respectively. None of the seven tumours with SPF above 12% showed an objective response to endocrine therapy, whereas 26/49 (52%) of those with lower SPF values responded ($P$ = 0.01). DNA ploidy, tumour grade, T-stage or M-stage did not significantly correlate with endocrine responsiveness. SPF was also the best predictor of progression free survival in patients treated hormonally. In conclusion, detection of high SPF in prostate cancer may indicate lack of hormonal responsiveness and poor prognosis.

Analysis of tumour DNA ploidy and S-phase fraction (SPF) by flow cytometry has been shown to provide additional independent information to prognostic assessment in a variety of malignancies (Merkel & McGuire, 1990; Hedley, 1989). Several studies indicate that prostate cancer patients with DNA-aneuploid tumours have shorter disease-free and overall survival than those with DNA-diploid tumours (Fordham *et al.*, 1986; Lee *et al.*, 1988; Montgomery *et al.*, 1990; Nativ *et al.*, 1989; Stephenson *et al.*, 1987; Winkler *et al.*, 1988). However, it is still controversial whether aneuploidy is an independent prognostic indicator. At least four groups have reported DNA aneuploidy to be an additional prognostic factor in prostate cancer (Adolfsson *et al.*, 1990; Montgomery *et al.*, 1990; Nativ *et al.*, 1989; Stephenson *et al.*, 1987), whereas some other investigators have failed to detect any independent value for aneuploidy after correcting for the effect of other prognostic indicators (Detjer *et al.*, 1989; Haugen & Mjölneröd, 1990; Ritchie *et al.*, 1988).

Few investigators have studied SPF from prostatic carcinomas. SPF appears to be higher in carcinomas than in benign prostate lesions (Schultz *et al.*, 1985) as well as in poorly differentiated carcinomas as compared to well-differentiated ones (Neill *et al.*, 1989). SPF has also been found to be higher in DNA-aneuploid tumours than in DNA diploid ones (Frankfurt *et al.*, 1984). However, the actual prognostic value of SPF in prostate cancer has remained unknown. In the present study we compared the significance of tumour DNA ploidy and SPF in the assessment of long-term prognosis and responsiveness to endocrine therapy in 78 primary prostatic carcinoma patients. A new software program was used to more accurately analyse SPFs from DNA histograms obtained from paraffin-embedded tissues (Kallioniemi *et al.*, 1991).

### Materials and methods

### *Patients*

The material consisted of 78 previously untreated histologically confirmed primary prostatic carcinomas diagnosed at the Tampere University Central Hospital during 1977–1979. The patients were staged using the TNM classification as recommended by UICC (UICC, 1987). The number of patients classified as T1, T2, T3 and T4 was 14, 16, 20 and 24, respectively. In four cases data on primary spread was not available. Twenty-six patients had distant metastases at the time of diagnosis (M1). Radical prostatectomy was done in four cases and four patients received a radical dose of radiation therapy. Three patients were treated with estramustine, whereas 60 received only hormonal therapy as the primary treatment. In the latter group the type of endocrine therapy was either orchiectomy (48 cases), oestrogen therapy (five cases) or orchiectomy with oestrogen therapy (seven cases). Disease progression was monitored by experienced urologists. Of the 60 patients who received endocrine therapy 50 (83%) were bone scanned. Serum levels of alkaline and acid phosphatases were also regularly measured in monitoring progression. Size of the prostate was primarily evaluated by digital examination, but also ultasonic measurement was sometimes used. Progression was defined either as (1) the increase of serum tumour marker levels more than 30% or over the upper reference value if tumour markers were normal at the time of diagnosis, (2) definite increase of primary tumour volume, (3) new findings in the bone scan or unexplained backache, (4) new ureteric obstruction, (5) new metastases in the lungs or in the urethra.

### *Tumour preparation and flow cytometric analysis*

Routinely stained slides of each tumour were examined by a pathologist who selected representative tissue blocks for DNA flow cytometry. The samples were Tru-Cut- needle biopsies in 41 cases, transurethral resection specimens in 28 cases and open surgical biopsies in nine cases. In case of transurethral resection specimens, only tumour-rich areas were used for flow cytometry. The histological typing and grading was evaluated by one experienced uropathologist according to the WHO histopathological grading of prostatic tumours (Mostofi, 1980). Fifty micrometer sections were cut from corresponding paraffin-embedded blocks. The sections were dewaxed using a modification of Hedley's method (Hedley *et al.*, 1983) as previously described (Kallioniemi, 1988; Vindelöv *et al.*, 1983). Briefly, the sections were washed twice with xylene and rehydrated in a sequence of 100%, 95%, 70% and 50% ethanol and finally washed twice in

Correspondence: T. Visakorpi.
Received 21 February 1991; and in revised form 29 April 1991.

distilled water. The sections were digested overnight with trypsin and stained with 50 µg ml$^{-1}$ ethidium bromide in a hypotonic detergent solution followed by RNAase treatment. The nuclear suspensions were filtered through nylon net (pore size 55 µm) and analysed with an EPICS C flow cytometer (Coulter Electronics Inc, Hialeah, FL) using 488 nm argon laser excitation. At least 10,000 nuclei from each specimen were scanned at a rate of 40–200 nuclei/s.

*Analysis and interpretation of DNA histograms*

DNA aneuploidy was considered to be present if two clearly separate peaks were found in the DNA histogram. The coefficient of variation (CV) of the G0/G1 peaks was 8.19 ± 2.05% (mean ± s.d.). The peak with the lowest mean channel number was defined as the DNA-diploid peak and was used as a reference in the calculation of DNA index. The definition of DNA aneuploidy in the tetraploid region (DNA index 1.80–2.20) was based on the presence of either (1) more than 15% cells in the tetraploid region of a DNA histogram with a diploid SPF less than 15% or (2) evidence of a proliferating tetraploid clone with G2/M-cells and/or S-phase cells in the octaploid/hypo-octaploid region of a DNA histogram. The latter criterion was not used if there was evidence of nuclear aggregation as indicated by the presence of a peak in the hexaploid region. We have chosen 15% as a cut-off limit since this limit has been systematically used in our laboratory for the analysis of other human malignancies. Our experience indicates that if cell cycle distributions are calculated from cell populations (like small tetraploid peaks) accounting for less than 15% of the total cell count, the interference by nuclear debris and aggregates makes the analysis very unreliable.

Cell cycle distribution was analysed using the Multicycle software program (Phoenix Flow Systems, San Diego, CA). Most of the paraffin-embedded tumours produced a DNA histogram with a predominantly flat debris distribution on the left side of the diploid G0/G1 peak. This debris is mostly caused by cleaved nuclei that are produced during specimen preparation (Kallioniemi *et al.*, 1991). The MultiCycle software models the background debris by assuming a random probability of any nucleus being cut into random-sized fragments during sample processing. The background signals are then numerically eliminated from the DNA histogram on a channel-by-channel basis. Any residual excess background at low histogram channels is fitted by a power function. After debris subtraction a zero-order Dean and Jett (Dean & Jett, 1974) model for cell cycle analysis was used. In aneuploid cases only the cell cycle of the aneuploid clone was registered. Interference caused by the S- and G2/M-phase cells of the diploid clone was accounted for either by modelling two proliferating cell cycles or by excluding the G2/M peak of the diploid clone from the analysis using a specific software option. SPF was considered inevaluable in four cases.

*Statistical analysis*

Statistical analyses of the clinicopathological and survival data were done using the BMDP Statistical Software Package (Dixon, 1981). The significance of survival differences between patient groups was evaluated using the Mantel-Cox test (BMDP1L). Clinicopathological correlations of DNA ploidy were analysed with the Pearson chi-square test (BMDP4F) and those of SPF by variance analysis (BMDP7D). For calculating relative risks a univariate Cox proportional hazard model (BMDP2L) was used.

### Results

Thirty-one (40%) of the 78 primary prostatic carcinomas were DNA-aneuploid. Of the 31 aneuploid cases 16 had a tetraploid DNA index (1.80–2.20). DNA aneuploidy was significantly associated with high tumour grade, large size of prostate and the presence of distant metastases (M1) at the time of diagnosis (Table I). The incidence of aneuploidy also tended to be higher in locally advanced tumours (stages T3–T4) and in tumours from older patients, but these trends did not reach statistical significance.

Median SPF of all 74 evaluable tumours was 4.2% with a mean of 6.4 ± 5.8% (± s.d.). DNA-aneuploid tumours had a higher SPF value (10.7 ± 7.2%) than DNA-diploid ones (3.9 ± 2.7%). High SPF was associated with poorly differentiated high-grade tumours, large size of prostate, locally advanced tumours (T3–T4) and the presence of distant metastases (MI) (Table I). There was also a trend for SPF to be higher in tumours from older patients.

Progression-free survival rate was shorter in patients with DNA-aneuploid tumours in comparison to those with DNA-diploid ones (Figure 1a). The same difference was observed in overall survival rates (Figure 1b). After correcting for intercurrent deaths the significance of this difference increased further (Figure 1c). The relative risk of tumour progression or patient death was about two times higher in DNA-aneuploid than in DNA-diploid cases. Patients with tetraploid tumours (DNA index 1.80–2.20) did not have significantly different survival as those with other types of aneuploidy.

SPF turned out to be a better prognostic factor than DNA ploidy. Using the median SPF (4.2%) as a cut-off value, prostate cancer patients with high SPF had a significantly shorter progression-free survival (Figure 2a), overall survival (Figure 2b) and survival corrected for intercurrent deaths (Figure 2c) as those with a low SPF. Ten-year survival rate corrected for intercurrent deaths in cases with below median SPF was 80% as compared with 11% in those with above median SPF. According to a univariate Cox regression analysis this difference was equivalent to a risk ratio of about 7.5. Overall survival rates of patients with below and above median SPF were 45% and 0%, respectively.

To further evaluate the prognostic value of SPF a stratified analysis was carried out using survival corrected for intercurrent deaths as the end-point (Table II). SPF had a higher prognostic impact in DNA-diploid than in DNA-aneuploid cases. Patients with distant metastases (M1) could not be subdivided according to SPF into different prognostic categories, whereas in cases without distant metastases at the time of diagnoses (M0) SPF was a strong prognostic factor. An association between high SPF and poor prognosis was also detected among the well (grade I) and moderately (grade II) differentiated tumours. SPF did not have additional prognostic value in grade III tumours as virtually all these cases had a high SPF value.

**Table I** Clinicopathological characteristics in prostatic carcinoma. Relation to DNA ploidy and SPF

| *Characteristic* | *Aneuploid (No./total)* | *Aneuploid (%)* | *SPF (mean ± s.d.)* |
|---|---|---|---|
| Grade I | 9/37[c] | 24 | 4.1 ± 3.4[b] |
| Grade II | 11/28 | 39 | 6.1 ± 4.4 |
| Grade III | 11/13 | 85 | 13.6 ± 8.5 |
| T1 | 3/14 | 21 | 4.1 ± 4.0[b] |
| T2 | 4/16 | 25 | 3.7 ± 2.5 |
| T3 | 7/20 | 35 | 6.0 ± 4.9 |
| T4 | 14/24 | 58 | 9.6 ± 7.3 |
| M0 | 14/52[b] | 27 | 4.4 ± 3.7[b] |
| M1 | 13/22 | 59 | 9.7 ± 7.0 |
| Clinical size of prostate: | | | |
| 1 | 1/10[a] | 10 | 3.6 ± 2.5[a] |
| 2 | 14/37 | 38 | 5.1 ± 4.1 |
| 3 | 12/24 | 50 | 9.1 ± 7.7 |
| 4 | 3/3 | 100 | 13.3 ± 6.6 |
| Age of patient: | | | |
| <65 | 5/16 | 31 | 3.8 ± 3.0 |
| 65–75 | 13/36 | 36 | 6.1 ± 5.0 |
| >75 | 13/26 | 50 | 8.3 ± 7.5 |

Statistical significance of association: [a]$P < 0.05$, [b]$P < 0.01$, [c]$P < 0.001$.

**Figure 1** Prognosis of patients with primary prostatic carcinoma ($n = 78$) according to the DNA ploidy of the tumour cells. **a**, progression-free survival; **b**, overall survival; **c**, survival corrected for intercurrent deaths. RR = relative risk.

**Figure 2** Prognosis of patients with primary prostatic carcinoma ($n = 73$) according to the SPF of the tumour cells. A single cut-off value (4.2%) was used to divide the patients in two groups of equal size. **a**, progression-free survival; **b**, overall survival; **c**, survival corrected for intercurrent deaths. RR = relative risk.

**Table II** The significance of SPF as a predictor of prostatic carcinoma survival in subgroups of patients defined by other prognostic indicators

| *Characteristic* | *n* | *10-year survival rate (%)* *Low SPF* | *High SPF* | *P-value (Mantel-Cox)* |
|---|---|---|---|---|
| All patients | 74 | 80 | 10 | 0.0000 |
| DNA diploid | 47 | 80 | 20 | 0.0017 |
| DNA aneuploid | 27 | 50 | 0 | 0.0395 |
| Grade I | 35 | 100 | 60 | 0.0031 |
| Grade II | 27 | 60 | 10 | 0.0248 |
| Grade III | 12 | 0 | 0 | 0.7226 |
| M0 | 49 | 80 | 40 | 0.0118 |
| M1 | 22 | 0 | 0 | 0.5152 |

A single SPF cut-off value was used to divide the patients in two groups. In DNA-diploid and aneuploid cases the cut-off values for SPF were the means of each group (3.9% and 10.7%, respectively). All the other comparisons were done using the median 4.2% SPF value.

Endocrine responsiveness could be monitored in all the 60 patients who were given endocrine therapy as the only primary treatment. There was no significant difference in the hormonal response rates between DNA-diploid and DNA-aneuploid cases (Table III). Neither did the grade of the tumour show a significant correlation with response rates. However, SPF levels correlated significantly with endocrine responsiveness. All seven patients with an SPF value higher than 12% did not show any measurable response to endocrine therapy (Table III), whereas over half of the patients with lower SPF values responded. If the median value (4.2%) was used as a cut-off for SPF the relation to endocrine response was less significant. However, even when using this cut-off value progression-free survival was significantly longer in hormonally treated patients with low as compared to high tumour SPF ($P = 0.0006$, 5.2-fold higher risk of death). If the tumour SPF was over 12% the median progression-free interval of patients under hormonal therapy was only 18 months.

### Discussion

High tumour proliferation rate as defined by flow cytometric SPF analysis was shown to predict poor response rate to endocrine manipulation, rapid disease progression and high mortality rate in primary prostate cancer. As compared to

**Table III** The response of prostatic carcinoma to primary hormonal therapy according to DNA ploidy, SPF, grade, primary extension (T) and metastasis (M) of the tumours

| *Characteristic* | *Progression or stable disease* *No.* | *Percent* | *Partial or complete remission* *No.* | *Percent* |
|---|---|---|---|---|
| All patients | 33 | 55 | 27 | 45 |
| DNA diploid | 21 | 57 | 16 | 43 |
| DNA aneuploid | 12 | 52 | 11 | 48 |
| Low SPF (<12%)[a] | 25 | 49 | 26 | 51 |
| High SPF (>12%) | 7 | 100 | 0 | 0 |
| Grade I | 14 | 58 | 10 | 42 |
| Grade II | 12 | 48 | 13 | 52 |
| Grade III | 7 | 64 | 4 | 36 |
| T1 and 2 | 10 | 55 | 8 | 45 |
| T3 | 11 | 58 | 8 | 42 |
| T4 | 11 | 52 | 10 | 48 |
| M0 | 20 | 54 | 17 | 46 |
| M1 | 12 | 55 | 10 | 45 |

[a]Pearson chi-square $P = 0.0110$.

DNA ploidy, which also previously has been found to predict disease outcome (Fordham *et al.*, 1986; Haugen & Mjölneröd, 1990; Lee *et al.*, 1988; Montgomery *et al.*, 1990; Nativ *et al.*, 1989; Stephenson *et al.*, 1987; Winkler *et al.*, 1988), SPF had a stronger impact and provided additional information on prognosis both among the DNA-diploid and DNA-aneuploid groups. Previously, only two small studies have been published on SPF analysis by flow cytometry in prostate cancer (Neill *et al.*, 1989; Frankfurt *et al.*, 1984). These publications did not include follow-up studies. They reported a correlation between SPF and DNA ploidy as well as between SPF and tumour grade, which we also were able to confirm in the present larger series.

Insensitivity to primary endocrine therapy was characteristic to a small subgroup of cases with very high (>12%) SPF levels. No other tumour parameter including ploidy, grade or stage correlated significantly with the degree of hormonal response. It is generally recognised that the prediction of responsiveness to endocrine therapy is difficult in prostate cancer (Schultze & Isaacs, 1986). On the basis of *in vitro* studies of prostate cancer cell lines it appears that a major factor contributing to endocrine responsiveness is the presence on a functional androgen receptor (Horoszewicz *et al.*, 1983). Although androgen receptor negative tumour cells have been described by immunocytochemistry (Demura *et al.*, 1988), there is no direct evidence of a correlation between steroid receptor status of prostate tumours and clinical response rates. It remains to be determined, whether the absence of a measurable hormonal response in the rapidly proliferating tumours is specifically due to the lack of androgen-mediated growth regulation and/or activation of alternative growth promoting mechanisms (Morris & Dodd, 1990). Tumour grade did not predict endocrine responsiveness suggesting that the effect of SPF was probably not related to the loss of differentiation of tumour cells. It should be emphasised that a number of prostatic carcinomas with low SPF also responded poorly or not at all to hormonal manipulation. Thus, tumour proliferation rate alone may not be a sufficient marker of endocrine responsiveness in prostate cancer but should be taken into account as a potential confounding variable in prospective trials on endocrine therapy.

High SPF had the strongest impact on prostate cancer survival among patients who did not have distant metastases at the time of diagnosis (M0-stage). Our patient material was still too small to definitely subdivide the M0 cases further according to T-stage and define the prognostic impact of SPF in such subgroups. However, preliminary data would indicate that SPF is a prognostic parameter in both early (T1–T2) and more advanced (T3–T4) non-metastatic tumours. Taken alone, SPF is about as powerful prognostic indicator than M-stage, T-stage or grade and appears to correlate even better with therapy response. However, SPF is significantly associated not only with the T- and M-stage, but also with grade and tumour ploidy, all established prognostic factors in prostate cancer. To better evaluate the exact value of SPF in determining prognosis and for guiding clinical treatment decisions in each prostate cancer subgroup we are currently evaluating a larger patient material. This would make it possible to perform multivariate regression analyses and to develop regression trees for guiding prognostic assessments.

Analysis of SPF by flow cytometry from tumours in general, and from paraffin-embedded material in particular, has been criticised due to lack of accuracy and reproducibility (Vindelöv & Christensen, 1990). However, DNA flow cytometry remains the best method available to access archival material and determine proliferation status of tumours from patients followed clinically for many years (Hedley, 1989). Futhermore, recent progress in the development of sophisticated computer models has made it possible to overcome some of the shortcomings in the methodology. It is nowadays possible to effectively discriminate the aneuploid SPF from overlapping diploid cell cycle as well as compensate for nuclear debris overlying the SPF in DNA histograms. We have recently demonstrated that the prognostic value of SPF is improved by subtracting the debris signals using a 'sliced nuclei' model that effectively eliminates most of the debris from archival tumour tissues underlying in DNA histograms (Kallioniemi *et al.*, 1991). We could reproducibly analyse cell cycle distributions from as many as 95% of the DNA histograms considered evaluable for DNA ploidy, which makes it unlikely that a selection bias could have affected our comparison of SPF and DNA ploidy as prognostic indicators (Hedley *et al.*, 1987).

However, it should be recognised that in DNA-diploid tumours it is still not possible to discriminate the cell cycle of tumour cells from that of stromal and inflammatory cells that are projected on the same channels in the DNA histogram. In the present study we tried to prepare tumour-rich areas from paraffin blocks for flow cytometric analysis. Since SPF values had better prognostic value in the DNA-diploid than in the DNA-aneuploid cases it is likely that at least the presence of an elevated SPF value in DNA-diploid tumours should be taken into account. Furthermore a correlation has been reported between flow cytometric SPF and $^3$H-thymidine labelling index (McDivvitt *et al.*, 1986) as well as between SPF and expression of proliferation antigens (Dawson *et al.*, 1990).

Our results indicate that determination of tumour cell proliferation by flow cytometric SPF analysis reflects the inherent biological aggressiveness and therapy responsiveness of prostatic carcinomas. Analysis of tumour proliferation rate may in the near future play a major role in improving prognostic and therapeutic assessments of patients. Although the subgroups that most likely would benefit from the analysis are not yet known, determination of tumour proliferation rate should be incorporated into randomised clinical trials on prostate cancer therapy.

This work was supported by Lahtikari Foundation. The authors thank Mrs Leena Pankko for technical assistance.

### References

ADOLFSSON, J., RÖNSTRÖM, L., HEDLUND, P.-O., LÖWHAGEN, T., CARSTENSEN, J. & TRIBUKAIT, B. (1990). The prognostic value of modal deoxyribonucleic acid in low grade, low stage untreated prostate cancer. *J. Urology*, **144**, 1404.

DAWSON, A.E., NORTON, J.A. & WEINBERG, D.S. (1990). Comparative assessment of proliferation and DNA content in breast carcinoma by image and flow cytometry. *Am. J. Pathol.*, **136**, 1115.

DEAN, P.N & JETT, J.H. (1974). Mathematical analysis of DNA distributions derived from flow microfluorometry. *J. Cell Biol.*, **60**, 523.

DEMURA, T., KUZUMAKI, N., ATSUSHI, O. & 6 others (1988). Establishment on monoclonal antibody to human androgen receptor and its clinical application for prostatic cancer. *Am. J. Clin. Oncol.* (Suppl.), **2**, S23.

DETJER, S.W., CUNNINGHAM, R.E., NOGUCHI, P.D. & 4 others (1989). Prognostic significance of DNA ploidy in carcinoma of prostate. *Urology*, **33**, 361.

DIXON, W.J. (1981). BMDP Statistical Software. Berkeley, Los Angeles. University of California Press: London.

FRANKFURT, W.R., GRECO, W.R., SLOCUM, H.K. & 4 others (1984). Proliferative characters of primary and metastatic human solid tumors by DNA flow cytometry. *Cytometry*, **5**, 629.

FORDHAM, M.V.P., BURDGET, A.H., MATTHEWS, J., WILLIAMS, G. & COOKE, T. (1986). Prostatic carcinoma cell DNA content measured by flow cytometry and its relation to clinical outcome. *Br. J. Surg.*, **73**, 40.

HAUGEN, O.A. & MJÖLNERÖD, O. (1990). DNA ploidy as prognostic factor in prostatic carcinoma. *Int. J. Cancer*, **45**, 224.

HEDLEY, D.W. (1989). Flow cytometry using paraffin-embedded tissue: five years on. *Cytometry*, **10**, 229.

HEDLEY, D.W., FRIEDLANDER, M.L., TAYLOR, I.W., RUFF, C.A. & MUSGROVE, E.A. (1983). Method for analysis of cellular DNA content of paraffin-embedded pathological material using flow cytometry. *J. Histochem. Cytochem.*, **31**, 1333.

HEDLEY, D.W., RUGG, C.A. & GELBER, R.D. (1987). Association of DNA index and S-phase fraction with prognosis of nodes positive early breast cancer. *Cancer Res.*, **47**, 4729.

HOROSZEWICZ, J.S., LEONG, S.S., KAWINSKI, E. & 5 others (1983). LNCaP model of human prostatic carcinoma. *Cancer Res.*, **43**, 1809.

KALLIONIEMI, O.-P. (1988). Comparison of fresh and paraffin-embedded tissue as starting material for DNA flow cytometry and evaluation on intratumor heterogeneity. *Cytometry*, **9**, 164.

KALLIONIEMI, O.-P., VISAKORPI, T., HOLLI, K., HEIKKINEN, A., ISOLA, J. & KOIVULA, T. (1991). Improved prognostic impact of S-phase values from paraffin embedded breast and prostate carcinomas after correcting for nuclear slicing. *Cytometry*, **12**, 413.

LEE, S.E., CURRIN, S.M., PAULSON, D.F. & WALTHER, P.J. (1988). Flow cytometric determination of ploidy in prostatic adenocarcinoma: a comparison with seminal vesicle involvement and histopathological grading as a predictor of clinical recurrence. *J. Urology*, **140**, 769.

McDIVITT, R.W., STONE, K.R., CRAIG, R.B., PALMER, J.O., MEYER, J.S. & BAUER, W.C. (1986). A proposed classification of breast cancer based on kinetic information. *Cancer*, **57**, 269.

MERKEL, D.E. & McGUIRE, W.L. (1990). Ploidy proliferative activity and prognosis. DNA flow cytometry of solid tumors. *Cancer*, **65**, 1194.

MONTGOMERY, B.T., NATIV, O., BLUTE, M.L. & 5 others (1990). Stage B prostate adenocarcinoma. Flow cytometric nuclear DNA ploidy analysis. *Arch. Surg.*, **125**, 327.

MORRIS, G.L. & DODD, J.G. (1990). Epidermal growth factor receptor mRNA levels in human prostatic tumors and cell lines. *J. Urol.*, **143**, 1272.

MOSTOFI, F.K. (1980). *Histological Typing of Prostate Tumours.* World Health Organization: Geneva.

NATIV, O., WINKLER, H.Z., RAZ, Y. & 5 others (1989). Stage D prostatic adenocarcinoma: flow cytometric nuclear DNA ploidy analysis. *Mayo Clin. Proc.*, **64**, 911.

NEILL, W.A., NORVAL, M. & HABIB, F.K. (1989). Nuclear DNA analysis of prostate tissue: correlation with stage and grade of tumour. *Urol. Int.*, **44**, 141.

RITCHIE, A.W.S., DOREY, F., LAYFIELD, L.J., LOVREKOVICH, J.H.H. & DEKERNION, J.B. (1988). Relationship of DNA content to conventional prognostic factors in clinically localised carcinoma of the prostate. *Br. J. Urol.*, **62**, 254.

SCHULTZ, R.E., VARELLO, M.A., TSOU, K.C., WEIN, A.J. & MURPHY, J.J. (1985). Simultaneous flow cytometric deoxyribonucleic acid and acid phosphatase analysis of benign and malignant lesions of the prostate. *J. Urol.*, **134**, 1133.

SCHULTZE, H. & ISAACS, J.T. (1986). Biology and therapy of prostatic cancer. *Cancer Surv.*, **5**, 487.

STEPHENSON, R.A., JAMES, B.C., GAY, H., FAIR, W.R., WHITMORE, W.F. Jr & MELAMED, M.R. (1987). Flow cytometry of prostate cancer: relationship of DNA content to survival. *Cancer Res.*, **47**, 2504.

UICC (1987). TNM. *Classification of Malignant Tumours.* Fourth Edition. Springer-Verlag: Berlin, Heidelberg, New York, London, Tokyo.

VINDELÖV, L.L. & CHRISTENSEN, I.J. (1990). A review of techniques and results obtained in one laboratory by an intergrated system of methods designed for routine clinical flow cytometric DNA analysis. *Cytometry*, **11**, 753.

VINDELÖV, L.L., CHRISTENSEN, I.J. & NISSEN, N.I. (1983). A detergent-trypsin method for the preparation of nuclei for flow cytometric DNA analysis. *Cytometry*, **3**, 323.

WINKLER, H.Z., RAINWATER, L.M., MYERS, R.P. & 4 others (1988). Stage D1 prostatic adenocarcinoma: significance of nuclear DNA ploidy patterns studied by flow cytometry. *Mayo Clin. Proc.*, **63**, 103.